# Rotator Cuff and Deltoid Involvement Are Associated with Shoulder Instability After Proximal Humerus Megaprosthetic Reconstruction

**DOI:** 10.3390/jcm15187006

**Published:** 2026-09-10

**Authors:** Luigi Cianni, Sara Martellini, Raffaele Vitiello, Alessandro El Motassime, Giulio Maccauro, Maristella Francesca Saccomanno

**Affiliations:** 1 Orthopaedics and Trauma Surgery Unit, Department of Ageing, Neurosciences, Head-Neck and Orthopaedics Sciences, Fondazione Policlinico Universitario Agostino Gemelli IRCCS, 00168 Rome, Italy; luigi.cianni1@guest.policlinicogemelli.it (L.C.); raffaele.vitiello@policlinicogemelli.it (R.V.); giulio.maccauro@policlinicogemelli.it (G.M.); maristellafrancesca.saccomanno@policlinicogemelli.it (M.F.S.); 2 Orthopaedics and Trauma Surgery Unit, Catholic University of the Sacred Heart, 00168 Rome, Italy; alessandro.elmotassime01@icatt.it

**Keywords:** proximal humerus reconstruction, megaprosthesis, postoperative shoulder instability, rotator cuff involvement, deltoid involvement, bone tumors, limb salvage surgery

## Abstract

**Background:** Reconstruction with megaprostheses is an important limb-salvage option in cases of bone tumors and metastases, as it allows extensive bone resection while preserving the limb. However, postoperative shoulder instability remains a frequent complication. Although implant instability is one of the leading causes of treatment failure, the factors contributing to it are still not clearly defined. This study analyzes the correlation between resection length, implant type, and involvement of the deltoid and rotator cuff in relation to postoperative instability and the risk of postoperative dislocation. **Methods:** Twenty-five patients treated at our institution between 2014 and 2025 who underwent proximal humerus replacement with a megaprosthesis were included. Patients were divided into two groups: those with postoperative dislocation (Group A) and those without (Group B). Functional outcomes were assessed using the MSTS and DASH scores. The mean follow-up was 20.38 ± 11.87 months. **Results:** Of the twenty-five patients included, six (24%) experienced instability and subsequent dislocation (Group A). Rotator cuff invasion was present in 100% of Group A and 36.8% of Group B (*p* < 0.001), while deltoid invasion was present in 83.3% of Group A and 21% of Group B (*p* = 0.001). The mean resection length was 14.4 ± 2.2 cm in Group A and 13.7 ± 3.7 cm in Group B (*p* = 0.636). No significant differences were found between the groups in DASH and MSTS scores. **Conclusions:** In this cohort, rotator cuff and deltoid involvement were significantly associated with postoperative shoulder instability following proximal humerus megaprosthetic reconstruction. These findings suggest that careful preoperative assessment of rotator cuff and deltoid involvement may help identify patients at higher risk of postoperative instability and optimize surgical planning. Future studies should focus on preoperative planning and the development of a risk score to identify patients at higher risk of instability, potentially allowing for additional procedures such as latissimus dorsi flap reconstruction.

## 1. Introduction

The proximal humerus is the fourth most common site for primary bone tumors, accounting for approximately 10–15% of osteosarcomas and 10% of Ewing sarcomas, and it is also the second most frequent site of metastatic bone involvement [1].

Tumors in this region are often associated with potential involvement of the glenohumeral joint, and reconstruction largely depends on both dynamic and static stabilizers of the shoulder. Limb-salvage surgery has become the treatment of choice following extensive resections in both primary bone tumors and metastatic disease [2]. In cases of significant bone loss, megaprostheses allow wide resection while preserving the limb; however, these reconstructions are associated with high complication rates [3].

Glenohumeral instability is consistently reported as one of the most common complications following proximal humeral megaprosthetic reconstruction and has been associated with worse functional outcomes [4]. In these patients, the soft tissue envelope is often compromised, and in many cases the deltoid insertion is partially detached. Wide resections require removal of a variable amount of surrounding normal tissue, including key stabilizing structures such as the rotator cuff. Preservation and reconstruction of the surrounding soft tissues are essential to ensure both good function and stability in the post-operative period [4,5].

Following resection of the proximal humerus, there are various reconstructive options available. Current reconstructive approaches include anatomical and reverse megaprostheses, allograft–prosthesis composites, osteoarticular allografts and biological reconstruction [6]. Hemiarthroplasty and allograft–prosthetic composite reconstruction have traditionally been considered the gold standard, especially in cases of limited bone loss, providing relatively low complication rates but often limited function below shoulder level [7]. More recently, reverse shoulder megaprostheses have been increasingly adopted for oncological reconstruction, particularly when rotator cuff deficiency is expected, relying on deltoid function to restore shoulder elevation [5,8].

Although implant instability is recognized as one of the most common causes of failure following proximal humerus megaprosthetic reconstruction, there is still no consensus regarding the main contributing factors. Resection length and preservation of the deltoid muscle may influence implant stability, but their exact roles remain unclear.

Recent systematic reviews have highlighted the heterogeneity of the available evidence regarding instability rates and reconstructive techniques, underscoring the need to better define the factors associated with postoperative instability [4].

The primary objective of this study was to evaluate the association between resection length, implant design, rotator cuff involvement, and deltoid muscle involvement and postoperative instability and dislocation following proximal humerus megaprosthetic reconstruction. The secondary objective was to evaluate functional outcomes using the Musculoskeletal Tumor Society (MSTS) and Disabilities of the Arm, Shoulder, and Hand (DASH) scores in patients with and without postoperative instability.

We hypothesized that rotator cuff and deltoid involvement would be associated with a higher rate of postoperative instability following proximal humerus megaprosthetic reconstruction.

## 2. Materials and Methods

### 2.1. Study Design and Patient Selection

The study was designed as a retrospective observational study and performed according to the PROCESS guidelines [9]. The study protocol was formally reviewed and approved by the Council of the Orthopaedics and Traumatology Institute of Fondazione Policlinico Universitario Agostino Gemelli IRCCS during its meeting held on 2 July 2026. As this was a retrospective observational study based exclusively on data previously collected during routine clinical care, no specific approval number was assigned as part of the internal institutional review process.

The study complied with the Declaration of Helsinki [10] and national ethical standards. According to institutional protocols, informed consent for surgery and for the collection of clinical data for scientific purposes was obtained from each patient at admission and prior to surgery. The inclusion criteria were a primary or metastatic lesion of the proximal humerus requiring reconstruction with a modular shoulder prosthesis, including established pathological fractures and lesions at risk of impending pathological fracture, and a minimum follow-up of six months.

In patients with metastatic disease without an established pathological fracture, a Mirels score ≥ 9 [11] was used to identify lesions at high risk of impending fracture and to support the indication for surgical treatment.

The exclusion criteria were non-pathological proximal humerus fractures, revision surgeries (either humeral nailing or shoulder prosthesis), traumatic shoulder dislocation, periprosthetic infection, patients younger than 18 years of age, or a minimum follow-up of less than six months.

Preoperative shoulder function assessment was not feasible due to patients’ general condition, pain, and the presence of fractures.

### 2.2. Surgical Technique

Antibiotic prophylaxis consisted of cefazolin 2 g administered intravenously preoperatively [12]. In patients with contraindications to cefazolin, intravenous vancomycin was administered according to the institutional protocol. A urinary catheter was placed in all patients and removed within 48 h. All procedures were performed under general anesthesia.

Patients were positioned in the beach-chair position, and proximal humerus resection was performed according to Enneking’s criteria [13]. A silver-coated modular prosthesis [14] was implanted in all cases. A lateral or deltopectoral approach was used. The choice of surgical approach was made preoperatively by the senior surgeons (M.F. Saccomanno and G. Maccauro) based on the evaluation of preoperative imaging, fracture characteristics, and individual surgical planning. Two prosthetic systems were used in this cohort: the MUTARS^®^ Proximal Humerus system (implantcast GmbH, Buxtehude, Germany) and the Equinoxe^®^ HRB (Exactech, Inc., Gainesville, FL, USA). The choice of reverse shoulder arthroplasty rather than conventional endoprosthetic reconstruction was individualized according to the treating surgeon’s clinical judgment, considering the patient’s clinical and oncological condition, expected survival, and the presence of pre-existing glenohumeral osteoarthritis when applicable. In each case, we aimed to preserve as much soft tissue as possible while ensuring adequate oncological margins.

In all patients, a Trevira tube^®^(implantcast GmbH, Buxtehude, Germany) [14] was used for soft-tissue reconstruction. When the rotator cuff and deltoid were not involved by the tumor, the preserved soft tissues were reattached to the Trevira tube. In cases of tumor involvement requiring resection of the rotator cuff and/or deltoid, the involved tissues were resected and were not reinserted; the remaining viable soft tissues were attached to the Trevira tube whenever possible. The axillary nerve was preserved in all patients, as no patient had tumor involvement of the nerve.

In patients with concomitant tumor involvement of the rotator cuff and deltoid muscle requiring additional soft-tissue reconstruction, a latissimus dorsi flap was performed in collaboration with plastic surgeons. The latissimus dorsi tendon was isolated and prepared, then tubularized for prosthetic coverage. Following humeral resection and megaprosthesis implantation, the latissimus dorsi flap was reinserted using a Trevira mesh (implantcast GmbH, Buxtehude, Germany).

### 2.3. Postoperative Rehabilitation

The postoperative protocol was as follows:

Immediate postoperative immobilization with an arm brace was maintained for one month. After the first postoperative month, rehabilitation was initiated with passive mobilization, including Codman pendulum exercises, for an additional month. Thereafter, cautious active mobilization was progressively introduced according to clinical evaluation. Clinical and radiographic evaluations were performed at 1 and 3 months, with subsequent follow-up at 6 and 12 months.

### 2.4. Clinical and Functional Assessment

Functional outcomes were assessed using the Musculoskeletal Tumor Society (MSTS) score [15], which was clinician-administered in English, and the validated Italian version of the Disabilities of the Arm, Shoulder, and Hand (DASH) questionnaire [16,17].

Postoperative instability was characterized using an adaptation of the glenohumeral instability classification described by Gerber et al. [18].

### 2.5. Statistical Analysis

Continuous variables were assessed for normality using the Shapiro–Wilk test. Normally distributed continuous variables were compared between groups using an independent-samples *t*-test, whereas non-normally distributed variables were analyzed using the Mann–Whitney U test. Categorical variables were compared using Fisher’s exact test because of the small sample size and expected cell counts. Statistical significance was set at *p* < 0.05.

## 3. Results

**Figure 1:**
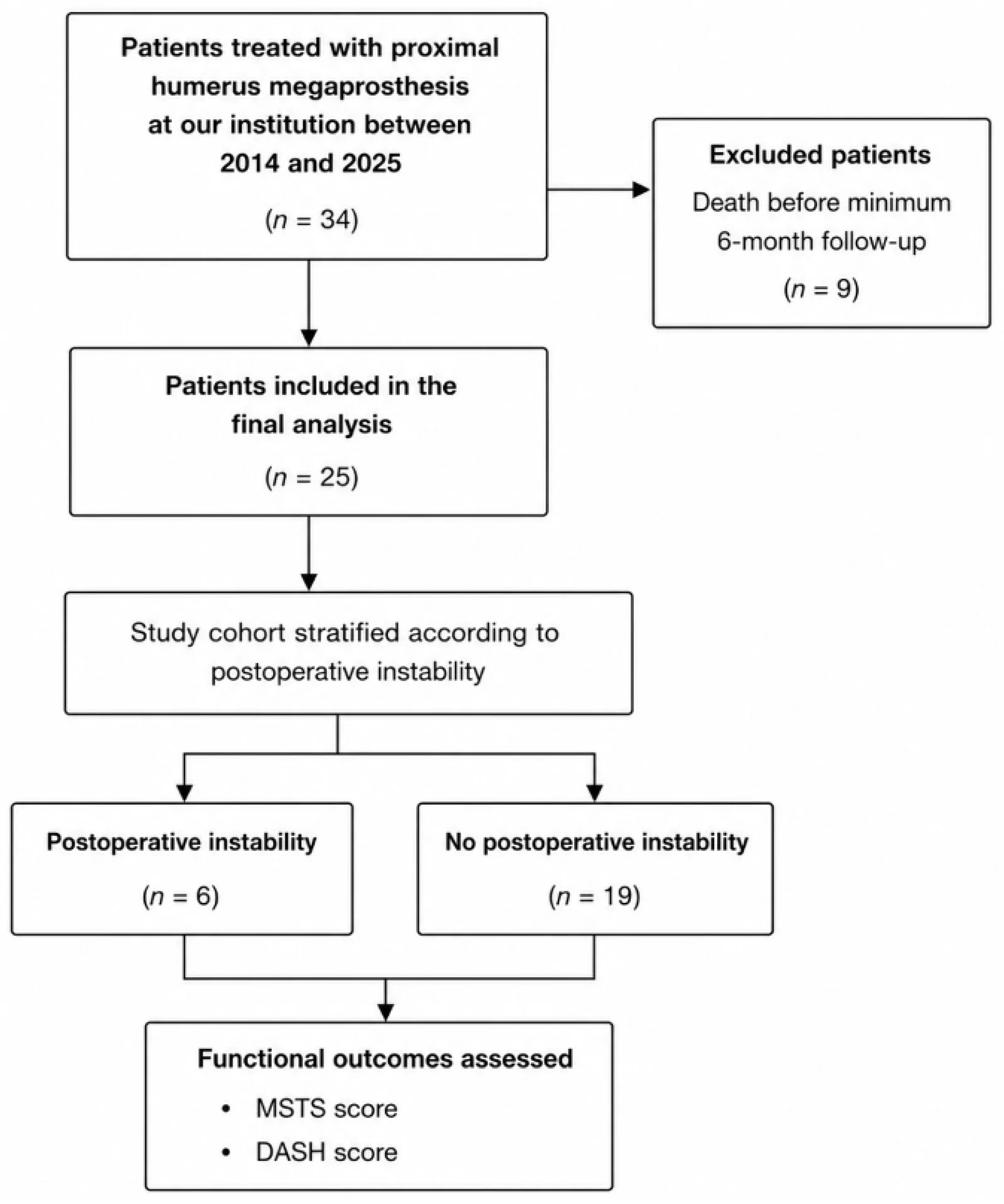
Patient selection.

Between 2014 and 2025, 34 patients underwent proximal humerus replacement with a megaprosthesis at our institution. Nine patients died before reaching the minimum follow-up of six months and were excluded. Therefore, 25 patients were included in the final analysis (Figure 1).

The most common tumor type was renal cell carcinoma *(n* = 7, 28%), followed by primary bone tumors (*n* = 6, 24%), breast cancer (*n* = 4, 16%), lung cancer (*n* = 3, 12%), brain tumors (*n* = 2, 8%), lymphoma (*n* = 2, 8%), and uterine cancer (*n* = 1, 4%). Among patients with metastatic disease, seven patients without an established pathological fracture were assessed using the Mirels score, with a mean score of 9.71 ± 1.11.

Regarding surgical treatment, the mean length of humeral resection was 13.87 ± 3.37 cm (range, 10–18 cm). One patient (4% of the overall cohort) underwent total humerus resection and replacement; this patient belonged to Group A and developed postoperative dislocation. The remaining 24 patients (96%) underwent proximal humerus reconstruction. Endoprosthetic reconstruction was performed in 21 patients (84%), while 4 patients (16%) underwent reverse shoulder arthroplasty. A lateral approach was used in 15 patients (60%) and a deltopectoral approach in 10 patients (40%).

At a mean follow-up of 20.38 ± 11.87 months, the overall mean MSTS score was 56.5 ± 18.1 and the overall mean DASH score was 46.7 ± 17.0.

Demographic and clinical characteristics and functional outcomes of the overall study cohort are summarized in Table 1, while the main surgical characteristics are reported in Table 2.

Postoperative dislocation occurred in 6 of 25 patients (24%), while 19 patients (76%) did not experience postoperative dislocation.

Postoperative infection occurred in 5 of 25 patients (20%), with a mean time to infection of 2.4 ± 1.7 months.

Patients were divided into two groups:

- Group A: patients with dislocation after humerus megaprosthesis;
- Group B: patients without dislocation;
- Group A included six patients (three females and three males), with a mean age of 64.17 ± 10.63 years;
- Group B included 19 patients (9 females and 10 males), with a mean age of 62.273 ± 13.83 years.

Baseline demographic and clinical characteristics are summarized in Table 3.

The mean MSTS score was 52.0 ± 16.4 in Group A and 57.9 ± 18.8 in Group B, with no statistically significant difference between the groups (*p* = 0.53). Similarly, the mean DASH score was 57.0 ± 17.3 in Group A and 43.5 ± 16.0 in Group B, also showing no statistically significant difference (*p* = 0.13).

To the authors’ knowledge, there is no established classification for shoulder instability in patients undergoing shoulder replacement, except for reverse shoulder arthroplasty. To better characterize instability in our cohort, we adapted the glenohumeral instability classification described by Gerber et al. [18].

According to this classification, instability in our patients can be described as dynamic, multidirectional instability without hyperlaxity.

The mean resection length was 14.4 ± 2.2 cm in Group A and 13.7 ± 3.7 cm in Group B (*p* = 0.636), with no statistically significant difference between groups. We analyzed soft tissue involvement using preoperative imaging (MRI and CT scans).

Rotator cuff invasion was observed in all patients who developed postoperative instability, whereas it was present in 36.8% of patients without instability (*p* < 0.001). Likewise, deltoid muscle invasion was more frequent in the instability group (83.3% vs. 21.0%; *p* = 0.001). Among patients without postoperative instability, three had concomitant involvement of both the rotator cuff and deltoid muscle; two of these patients underwent additional soft-tissue reconstruction with a latissimus dorsi flap.

Four patients underwent reconstruction with reverse shoulder arthroplasty (one in Group A and three in Group B), while twenty-one patients received an endoprosthesis (Table 4). The surgical approach was deltopectoral in 10 patients and lateral in 15 patient.

Postoperative infection occurred in 2 of 6 patients in Group A (33.3%) and in 3 of 19 patients in Group B (15.8%). The mean time to infection was 1.0 ± 0.0 months in Group A and 3.3 ± 1.5 months in Group B. Postoperative complications are summarized in Table 5.

**Figure 2:**
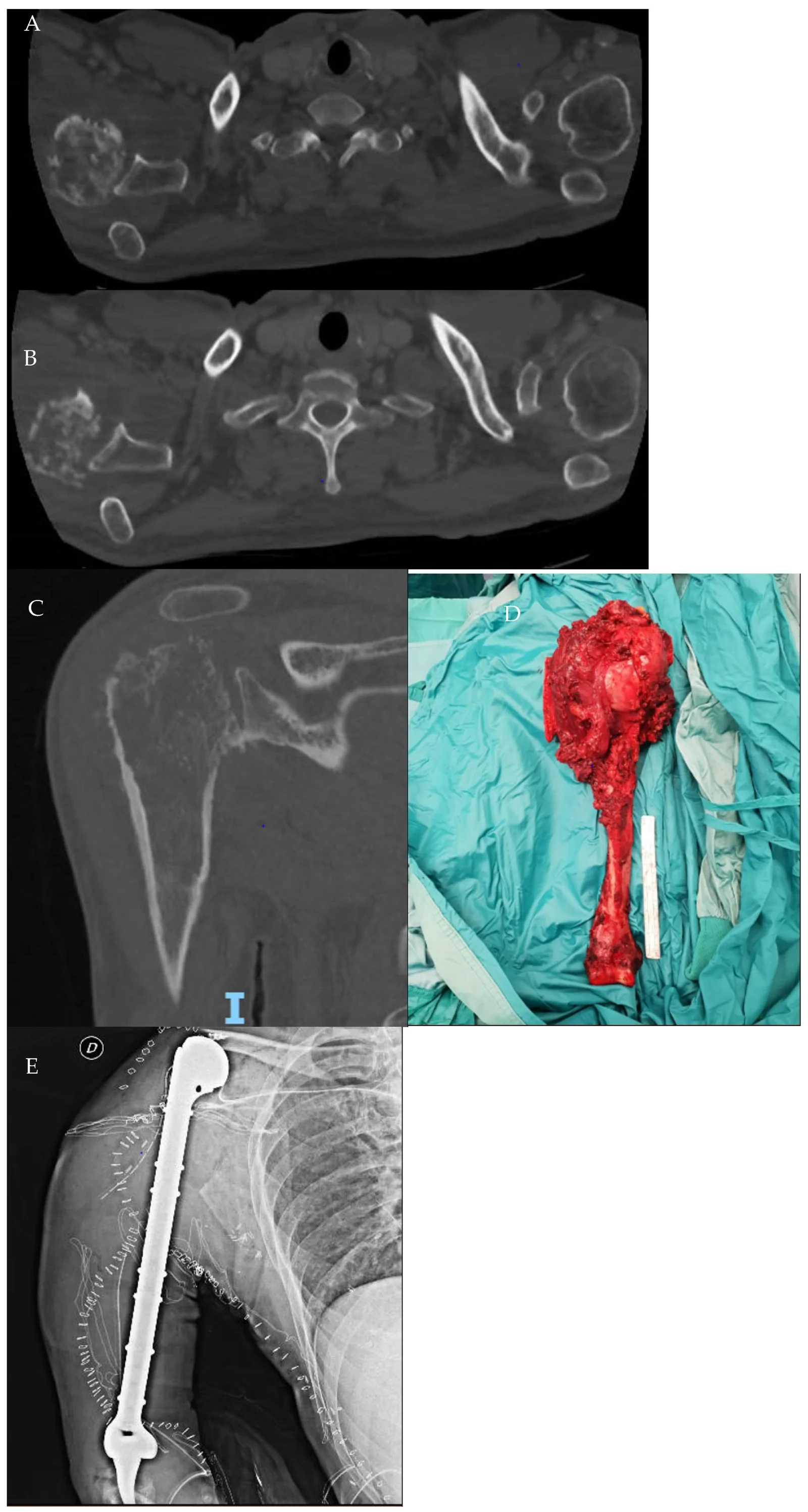
(**A**,**B**) Axial CT scan views; (**C**) coronal CT scan view; (**D**) total humerus resection for chondrosarcoma in a patient undergoing extra-articular resection; (**E**) postoperative X-ray showing total humerus replacement with an endoprosthesis in a patient treated with a latissimus dorsi flap.

Relevant images from one of these cases are shown in Figure 2A–E.

## 4. Discussion

The main finding of the present study was that rotator cuff and deltoid involvement were significantly associated with postoperative shoulder instability following proximal humeral megaprosthetic reconstruction. In contrast, resection length and implant type were not significantly associated with postoperative instability. Although patients who developed dislocation showed worse mean DASH and MSTS scores, these differences did not reach statistical significance. These findings support the relevance of the residual soft-tissue envelope in maintaining shoulder stability after proximal humeral reconstruction. However, given the small number of instability events, these associations should be interpreted with caution and cannot be considered independent predictors of postoperative dislocation.

Surgical treatment of bone metastases and primary bone tumors of the humerus may be indicated in patients with pathological or impending fractures [19]. Proximal humeral resection may require complex reconstruction, with several reconstructive options available depending on the extent of the resection and the structures involved, including megaprosthetic reconstruction, composite prostheses, allografts, arthrodesis, intramedullary fixation, and joint resection [20]. Despite advances in reconstructive techniques, postoperative instability remains an important complication following proximal humeral reconstruction. Angelini et al. reported a high rate of complications after extra-articular shoulder resections, with soft-tissue failure representing the most frequent complication [21]. De Geyer et al. also reported a relevant complication rate in patients undergoing surgery for humeral metastases, although their population mainly included intramedullary nailing and plate fixation and only a limited number of prosthetic reconstructions [22]. More recently, El Motassime et al. evaluated shoulder instability following proximal humeral reconstruction and reported subjective severe instability in approximately 60% of patients [23]. In that study, patients reporting instability also showed significantly worse DASH, MSTS, and WOSI scores. However, instability was evaluated using a patient-reported outcome measure, whereas the present study considered objectively documented postoperative dislocation [23].

Tong et al. investigated soft-tissue reconstruction using a ligament augmentation and reconstruction system (LARS) following proximal humeral resection [24]. Their study included both benign and malignant tumors and did not specifically evaluate tumor involvement of the rotator cuff and deltoid [24]. Therefore, although satisfactory outcomes were reported, direct comparison with the present study is limited. Similarly, Messina et al. identified soft-tissue failure—including painful instability, subluxation, and dislocation—as an important complication following megaprosthetic reconstruction of the proximal humerus and emphasized the importance of soft-tissue management [25]. Daher et al. [26] compared allograft–prosthesis composite reconstruction with megaprosthetic reconstruction. Megaprosthetic reconstruction was associated with a lower reoperation rate, whereas allograft–prosthesis composite reconstruction achieved higher MSTS scores and greater forward flexion. These findings suggest that reconstructive strategy may influence functional outcomes and should be considered together with the management of the surrounding soft tissues.

The choice of reconstruction after proximal humeral tumor resection should take into account both the extent of bone resection and the status of the remaining soft tissues. Several reconstructive options have been described, including anatomical megaprostheses, reverse shoulder arthroplasty, and allograft–prosthesis composite reconstruction [20]. In our cohort, implant design was not significantly associated with postoperative instability, with no statistically significant differences between endoprosthesis and reverse shoulder arthroplasty. However, given the limited number of patients treated with reverse shoulder arthroplasty, no definitive conclusions can be drawn regarding the influence of implant design on postoperative stability.

In our cohort, three patients with concomitant rotator cuff and deltoid involvement did not develop postoperative instability, and two of these patients underwent latissimus dorsi flap reconstruction. Although this observation may suggest a potential role for additional soft-tissue reconstruction in selected cases, the very small number of patients treated with this procedure precludes any conclusion regarding its effect on postoperative stability. Endoprosthetic suspension has also been described as an alternative strategy to improve shoulder stability after proximal humeral tumor resection. Fujibuchi et al. described a technique in which the humeral endoprosthesis was suspended from the acromion using polypropylene mesh; no instability was observed in the suspension group, despite abductor muscle resection in six of nine patients [27].

This study has several limitations. First, its retrospective, single-center design and small sample size, with only six postoperative instability events, limit the strength and generalizability of the findings. Due to the retrospective nature of the study, standardized data on glenoid orientation and component angulation were not available, precluding their evaluation as potential factors associated with postoperative instability. The study population was also heterogeneous in terms of diagnosis, reconstructive procedure, surgical approach, and extent of soft-tissue involvement. In addition, the relatively short and variable follow-up and the lack of preoperative functional scores limited the assessment of functional outcomes over time. Finally, the limited number of instability events precluded robust multivariable analysis; therefore, the observed associations cannot be interpreted as independent predictive effects.

## 5. Conclusions

Reconstructive surgery with proximal humeral megaprostheses represents a valid treatment option for patients with primary bone tumors or metastatic lesions, particularly in patients presenting with pathological fractures or lesions at high risk of fracture and with a favorable life expectancy. Despite its limitations, this study found that postoperative instability was associated with rotator cuff and deltoid involvement in this cohort, mainly due to the involvement of anatomical structures that normally stabilize the shoulder, even when these are partially reconstructed during surgery.

Careful preoperative assessment of soft-tissue involvement may help identify patients at increased risk of postoperative instability and guide the consideration of additional reconstructive procedures, including latissimus dorsi flap reconstruction, in selected patients with extensive soft-tissue involvement.

## Figures and Tables

**Table 1:** Demographic and clinical characteristics of the overall study cohort.

Characteristic	Overall Cohort
Total population	25
Age, years, mean ± SD	62.2 ± 11.95
Sex, *n* (%)	
Female	12 (48%)
Male	13 (52%)
Diagnosis, *n* (%)	
Renal cell carcinoma	7 (28%)
Primary bone tumors	6 (24%)
Breast cancer	4 (16%)
Lung cancer	3 (12%)
Brain tumors	2 (8%)
Lymphoma	2 (8%)
Uterine cancer	1 (4%)
Follow-up, months	20.38 ± 11.87

**Table 2:** Surgical characteristics.

Surgical Characteristic	Overall Cohort (*n* = 25)
Length of resection, cm	
Mean ± SD	13.87 ± 3.37
Range	10–18
Type of resection, *n* (%)	
Proximal humerus	24 (96%)
Total humerus	1 (4%)
Implant type, *n* (%)	
Endoprosthesis	21 (84%)
Reverse shoulder arthroplasty	4 (16%)
Surgical approach, *n* (%)	
Lateral	15 (60%)
Deltopectoral	10 (40%)
Axillary nerve, *n* (%)	
Preserved	25 (100%)
Sacrificed	0 (0%)

**Table 3:** Demographics and clinical characteristics of the study population.

Characteristic	Group A (*n* = 6)	Group B (*n* = 19)
Total population	6	19
Gender		
Female	3 (50%)	9 (47.4%)
Male	3 (50%)	10 (52.6%)
Diagnosis		
Primary bone tumors	1 (16.7%)	5 (26.3%)
Metastases	5 (83.3%)	14 (73.7%)

**Table 4:** Comparison of surgical and clinical variables between groups.

Variable	Group A (*n* = 6)	Group B (*n* = 19)	*p*-Value
Total population	6	19	—
Rotator cuff invasion	6 (100.0%)	7 (36.8%)	<0.001
Deltoid muscle invasion	5 (83.3%)	4 (21%)	0.001
Length of resection (cm)	14.4 ± 2.2	13.7 ± 3.7	0.636
Implant type			1.000
Reverse shoulder arthroplasty	1	3	—
Endoprosthesis	5	16	—
Dislocation time (months)	8.5 ± 10.61	—	—

**Table 5:** Postoperative complications in the study cohort.

Complication	Overall Cohort (*n* = 25)	Group A (*n* = 6)	Group B (*n* = 19)	Time to Complication, Months
Dislocation	6 (24.0%)	6 (100.0%)	0 (0.0%)	8.5 ± 10.6
Infection	5 (20.0%)	2 (33.3%)	3 (15.8%)	2.4 ± 1.7

## Data Availability

The original contributions presented in this study are included in the article. Further inquiries can be directed to the corresponding author.
